# Quality evaluation of the Azithromycin tablets commonly marketed in Adama, and Modjo towns, Oromia Regional State, Ethiopia

**DOI:** 10.1371/journal.pone.0282156

**Published:** 2023-03-02

**Authors:** Yesuneh Tefera Mekasha, Belachew Umeta Chali, Anbessa Bekele Feissa, Gemmechu Hasen Godena, Hassen Kebede Hassen, Sultan Suleman Wega

**Affiliations:** 1 Pharmaceutical Sciences, Pharmaceutical Quality Assurance and Regulatory Affairs, University of Gondar, Gondar, Ethiopia; 2 Pharmaceutical Sciences, School of Pharmacy, Institute of Health, Jimma University, Jimma, Ethiopia; 3 Veterinary Drug and Feed Control and Administration Authority, Addis Ababa, Ethiopia; Siksha O Anusandhan University School of Pharmaceutical Sciences, INDIA

## Abstract

**Background:**

Azithromycin is a therapeutically” relevant macrolide antibiotic registered on the Essential Medicines List of the World Health Organization. The fact that medicine is selected as an essential drug doesn’t mean that it is of good quality. Hence, a continuous quality evaluation of the drug should be mandated to verify that the right medication is available on the market.

**Objective:**

To evaluate the quality of Azithromycin Tablets commonly marketed in Adama, and Modjo town, Oromia Regional State, Ethiopia.

**Methods:**

All six brands were subjected to *in-vitro* quality control tests, which were carried out according to procedures described in the manufacturer’s method, the United States Pharmacopeia, and the WHO inspection tool. All quality control parameters were compared by one-way ANOVA. Statistically, significant difference was considered when P<0.05. The *in-vitro* dissolution profiles of the brands were also compared statistically using the post-hoc Dunnett test, model-independent and model-dependent approaches.

**Results:**

All of the evaluated brands agreed with WHO visual inspection criteria. All of the tablets achieved the thickness, and diameter test requirements of the manufacturer’s specification (±5%). All brands passed the hardness, friability, weight variation, disintegration, identity, and assay tests as stipulated by USP. The dissolution rate was more than 80% in 30 minutes, which was within the USP specification. The model-independent parameters have confirmed that only two brands (2/6) were deemed better brands for interchangeability. Weibull and Korsemeyer’s Peppas model were the best release models.

**Conclusion:**

All of the evaluated brands passed the quality specification. The model dependent approaches revealed that drug release data fit well to the Weibull, and Korsemeyer’s Peppas release models. However, the model-independent parameters have confirmed that only two brands were deemed better brands (2/6) for interchangeability. Due to the dynamic nature of low-quality medications, the Ethiopian Food, and Drug Authority should keep an eye on marketed products to guarantee their quality, especially for drugs like azithromycin for which non-bioequivalence data from the study has revealed a clinical concern.

## Introduction

Azithromycin is a macrolide that has a large lactone ring, which prevents the bacterial cells from synthesizing essential protein [1]. The drug is initially used to against infection caused by respiratory pathogens [2–4]. It is also used to treat bacterial infections like sexually transmitted diseases, as well as infections of the ears, sinuses, skin, throat, and reproductive organs [5]. Its chemical formula is C38H72N2O12.2H202, and its chemical structure consists of 15-membered rings with two deoxy-sugars. It is derived from erythromycin through a methyl-substituted nitrogen atom within the lactone ring and has a relative molecular mass of 748.98 g/mol [6].

Azithromycin is a low soluble, highly permeable antibiotic classified as class II by the Biopharmaceutical Classification System (BSC). The number of drug candidates that are poorly water soluble has lately surged in drug development [7]. Because the dissolving rate is insufficient to completely dissolve the medicine in the gastro intestinal tract. Its limited solubility causes poor and variable oral absorption. As a result, dissolution is the rate-limiting step in drug absorption. So that *in-vitro in-vivo* correlation (IVIVC) could be expected [8].

Azithromycin is therapeautically relevant macrolide antibiotic registered in essential medicines list of World Health Organization. It contains the safest and best medications to meet a key need in the healthcare system [9]. The fact that medicine is selected as an essential drug doesn’t mean that it is of good quality. Hence, a continuous quality evalaution of the drug should be mandated to verify that the right medication is available in the market. Pharmaceutical items are not ordinary foods, thus their quality must be regulated before and after they leave the manufacturing site, as well as at the point of entry, because they have a social, economic, and public health impact [10].

There is considerable up-to-date interest in the determination of the burden of a pandemic complaint called COVID-19, which has had a catastrophic effect on the world’s demographics, resulting in more than 3.8 million deaths worldwide [11]. The treatment of choice for this new pandemic remains unknown, so effective and safe treatments must be found urgently. The study showed that azithromycin was extensively estimated for its efficacy against COVID-19, and was highly rated [12–14]. To counteract the pandemic, the mass production of azithromycin proliferated the pharmaceutical market owing to its high consumption rates [15, 16]. Surprisingly, there has been a quality issue with azithromycin drugs, which are commonly used to treat life-threatening infections. This activity caught the attention of professionals due to the scarcity of quality pharmaceutical items within the market. There was evidence that a significant portion of azithromycin consumed around the world was of poor quality. According to a quality survey conducted in Cameroon in 2017, tested azithromycin samples deviated from the dissolution standard by the indicated amount(31.6%) [17]. Tie *et al*. from Belgium reported that tested azithromycin drugs failed the dissolution specification [18]. Furthermore, the survey performed by Egbo H in Nigeria, and Ghana revealed, a total of 68% of deviated from the label claim of the percentage drug content [19].

The drug regulatory authorities of the central laboratory (EFDA) may be unable to complete all of its accreditation for ensuring the quality of drugs procured and processed in the country. As a result, some illegal actions take place in the country, taking advantage of weaknesses in the legal system [20]. Also, the registration process and informal drug infiltration make surveillance measures even more difficult in Ethiopia [21]. Adama and Modjo towns, in particular, serve as commercial crossroads from the East Shoa zone. They are hubs of pharmaceutical activity as well as high costs associated with drug wastage due to expiration dates, poor storage conditions, and insufficient inventory management of vital medicines, particularly antibiotics were discovered [22].

The study conducted by Demissie et al. (2021) in Adama town revealed azithromycin was one of the most frequently prescribed antibiotics, accounting for 26.3% of local purchases and 73.7% from foreign regions. Moreover, two-thirds of the drug (68.4%) failed the efficacy test. Also, the majority of individuals in this town (92.9%) are unaware of the dangers of counterfeit antibiotics [23]. In the town of Modjo, there was no prior evidence regarding the status of the candidate drug. This survey has sparked an interest in the quality evaluation of azithromycin in the respective towns. Taking the aforementioned issue into consideration, the current study aims to evaluate the quality of the different brands of azithromycin tablets commonly marketed in Adama, and Modjo towns in East Shoa Zone, Oromia Regional State, Ethiopia.

## Materials and methods

### Study area and period

The samples were collected from Adama and Modjo towns in the East Shoa zone, Oromia regional state, Ethiopia. The experimental part of the study was undertaken in the Ethiopian Pharmaceutical Company Drug Quality Control Laboratory (EPHARM) from July to September 2021. Adama town is located 99 km southeast of Addis Ababa [24]. According to the Adama town health office report, the town has eight health centers (n = 8), one hospital pharmacies (n = 1), forty one private pharmacies (n = 41), sixty eight drug stores (68), twenty three wholesalers (n = 23), and fifty five clinics (n = 55) were found that serve a total population of 411,000 residents [25]. Modjo town is located 72 km at South East of Addis Ababa [26]. According to Modjo health office report the town has one hospitals (n = 1), twelve private drug retail stores (n = 12), two government health centers (n = 2), and fifteen clinics (n = 15) were in task that offered health services to a total population of 58,406 inhabitants, with 28083 male (48.1%) and 30,323 female (51.9%) [27].

### Instruments

HPLC (Mode: LC2030C3D, Schimadzu Corporation, Japan), analytical balance (Metler Toledo, Switzerland), pH meter (Metler Toledo, China), hardness, Thickness and Diameter tester (S.No.TDH-311, Pharma test, German), friability tester (Sotax, Switzerland), Disintegration time tester (ERWEKA, Germany), dissolution tester (Paddle, S.No. DT13460835, India), Sonicator (Bandelin, Germany), volumetric-flask (England), and 0.45μm Nylon membrane filter (Germany) were used for the study.

### Chemicals and reagents

Distilled water (EPHARM, Ethiopia), 900ml phosphate buffer (Batch.No.V4D440134L, Carlo Erba reagent, France, Assay = 99–100.55), Dibasic potassium phosphate (Batch.No.V4F6891041, Carlo Erba reagent, France), Sodium 1-octanesulfonate HPLC ion pairing reagent (Batch.No.8663154, Sisco research laboratory pvt.Ltd, India), Acetonitrile HPLC grade (Batch.No.AN647, Alpha chemical, India), Methanol HPLC grade(B.No.V4B459144B, Carlo Erba reagent, France), phosphoric acid (B.No.A215281612, Loba chemical pvt.Ltd, India, Assay = 99.0%), and Azithromycin working standard(potency = 99.8%, WS/20010) acquired from cadila pharmaceutical plc., Addis Ababa, Ethiopia), were used.

### Sampling techniques and sample collection

Quality evaluation test was conducted as per WHO sampling strategy [28]. Accordingly, a convenience sampling technique was used in the selection of the drug retail outlets while a covert (mystery shopper approach) sampling technique was considered for sample collection. Only Six brands of Azithromycin tablets were found at the time of the survey were collected from the hospital and the privately held drug retail outlets by mystery shopper approach.

The mystery shoppers were blinded to the study’s purpose and were trained for two days prior to sample collection (S1 File). If drug outlets had more than one brand, the mystery shopper selected all of the different brands by random selection of the drugs through a hand-written note with random numbers for varied numbers of brands to prevent seller bias.

Two mystery shoppers and one supervisor per drug retail outlets were assigned for sample collection. To sample, the mystery shopper pretended to be a patient or stated that he or she needed to send azithromycin brands to his or her relatives in a remote area where access to a health institute was limited. Other shoppers were presented with a prescription paper that was recommended by a physician to take azithromycin for severe (atypical) pneumonia infection. They mimicked, and asked further, "Do you have other azithromycin drugs that are cheaper?" Immediately after leaving the drug retail outlets where the individual brands were procured, all relevant information about the retrieved samples was recorded in the data sheet. Finally, the investigator double-checked the quality of the data.

Due to the absence of azithromycin innovator product on the Ethiopian pharmaceutical market, one of the brands tested (A6*) was selected as the comparator product. The comparator product was selected based on the WHO Guideline on the Selection of Comparator Pharmaceutical Products for Equivalence Assessment of Interchangeable Multisource (Generic) Products, which reveals the choice of a product that has been granted approval in an ICH-associated countries and market leader product was selected as a comparator [29].

A sample collection form was designed to enable the collection of samples from the field and structured to collect the following information per sample; place of sample collection, site of sample collection, country of manufacture, name of the medicine, dosage form, package size, Batch/lot number, brand name, label claim, manufacturing, and expiry date (S2 File).

All brands included in the study were registered by Electronic Regulatory Information system of Ethiopian Food, and Drug Authority.

The collected samples were transported to Ethiopian Pharmaceutical Drug Quality control Laboratory, and stored under storage condition specified on the label of the product until analysis. The detailed label information of different brands of Azithromycin 500 mg tablets collected from the respective towns were depicted in Table 1.

**Table 1:** A detailed label information of different brands of Azithromycin 500 mg tablets evaluated in the study area.

Code	Brand	Manufacturer	Country of origin	Batch. No	Mfg. date	Expiry date	Color	Shape
**A1**	Swazi	Indian swift limited	India	ACS10221	Jan.2021	Dec.2023	White	Oblong
**A2**	Zycin	Cadila pharmaceutical plc.	Ethiopia	D21001T355	June.2021	May.2024	White	Oblong
**A3**	Azeescot	Scott-Edil pharmaceutical	India	2115028T001	13/02/2021	12/02/2024	White	Oblong
**A4**	Azitro	Deva holding	Turkey	A087485	08/2020	07/2023	White	Oblong
**A5**	Zithrocin	Unique pharmaceutical laboratories	India	PZZ21003	Feb.2021	Jan.2024	Blue	Oblong
A6*	Zithrotel	Anfarm hellas	Greece	21C080	02/2021	02/2024	Yellow	Oblong

**Note**: A6* = Comparator product

### Quality evaluation parameters

All the six different brands of Azithromycin tablets collected from Adama, and Modjo town of East Shoa Zone were assessed for *in-vitro* quality control parameters like; visual inspection, thickness, diameter, hardness, friability, weight variation, disintegration, identity, assay, and dissolution test. The study was done according to method specified in the manufacturers method, United States Pharmacopeia, and WHO visual inspection tool.

#### Physical characteristics, packaging and labeling

The collected samples were visually inspected for their physical characteristics (shapes, color, breaks, marking, cracks, and splits), packaging, and labelling of information (name of API, the country of origin, manufacturing company, storage information, manufacturing date, expiry date, lot number, number of units per strip/packages, and medicine strength (mg/unit) as per the WHO designed checklist (S3 File) for healthcare professionals to carry out visual inspection of medicines for the signs of counterfeiting [30].

#### Thickness and diameter of tablets

Five tablets randomly selected and measured. Then, the standard deviation of each tablet from the batch calculated and compared with the specification [31].

#### Hardness test

The hardness of tablets was determined by selecting five tablets randomly from each batch. Each tablet was placed between two the anvils and force was applied to the anvils, and the crushing strength that causes the tablet to break were recorded [32].

#### Friability test

Twenty tablets were selected randomly, and carefully dedusted before testing. Then accurately weighed and placed in the drum of the friability tester, and rotated at 100 revolutions (25rpm for 4 minute). Remove any loose dust from the tablets as before, and again accurately weighed. Then, the percentage of weight loss was calculated using Eq (1), and compared with USP40-NF35 acceptance limits [33]. USP recommended that the percentage loss not more than 1% is considered to be acceptable and if cracked, cleaved, or broken tablets are present within the tablet sample after tumbling, the tablet fails the test.


$$\%\text{weight}\;\text{loss}=\frac{\text{Initial}\;\text{weight}-\text{Final}\;\text{weight}}{\text{Initial}\;\text{weight}}\times 100$$ (1)

#### Weight variation test

Twenty tablets from each brand were selected randomly, and individually weighed with the help of electronic balance. The individual weight of tablets were determined, and compared with the average weight. The percentage weight variations of the tablets were calculated using Eq (2). Tablets pass the USP standard if NMT two tablets were outside the percentage limit, and if no tablets differ by more than two times the percentage limit [34].


$$\%\text{Weight}\;\text{variation}=\frac{\text{Individual}\;\text{weight}-\text{Average}\;\text{weight}}{\text{Average}\;\text{weight}}\times 100$$ (2)

#### Disintegration test

The disintegration medium was filled with 800ml of distilled water maintained at 37±2°c. Then one tablet was placed in each of the tubes. The time required for each tablet to disintegrate was calculated.

#### Identification test

The identification test was carried out by comparing the retention time of the peak of azithromycin in the drug sample with that of the Working standard [34].

#### Assay of active ingredients

*Preparation of solution A*. Dibasic potassium phosphate about of 4.4g, and 0.5g of sodium1-octanesulfonate were mixed and transferred to a 1000ml of volumetric flask, and diluted with water to a required volume. Then the solution was adjusted with phosphoric acid to a pH of 8.20±0.05.

*Preparation of mobile phase*. The mobile phase was prepared by filtering and degassing the mixture of acetonitrile, methanol, and solution A in the ratio of 9: 3: 8, respectively.

*Preparation of diluent A*. Firstly, solution B was made by pouring around 1.7g of monobasic ammonium phosphate into a 1000ml volumetric flask and diluted with water to the desired level. The PH of the solution was adjusted with ammonium hydroxide to 10.00±.0.05. Finally, diluent A was made by mixing methanol, acetonitrile, and Solution B in the following proportions; 7:6:7 respectively.

*Preparation of standard solution*. First of all, working standard of an azithromycin solution was prepared by taking 20 mg of USP azithromycin working standard in a 50 ml volumetric flask. Then Diluent A was added up to the mark and sonicated to obtain a solution having a known concentration of 0.4 mg of azithromycin per ml.

*Preparation of sample solution*. Twenty tablets from each brand were randomly selected for the preparation of the sample solution. Then, the samples were weighed and finely powdered. An accurately weighed portion of a powder, equivalent to 667 mg of Azithromycin was transferred to a 200 ml volumetric flask. Then, diluent A was added to the required volume and sonicated for not less than 15 minutes. Then the solution is allowed to equilibrate to room temperature and diluted with Diluent A to the required volume and mixed to obtain a solution having a concentration of 0.4 mg of azithromycin per ml and passed through a filter having a porosity of 0.45 m. This method was used to make sample solutions for all commercially available Azithromycin tablet formulations that were under investigation. The chromatographic system for azithromycin working standard, and samples were displayed under Table 2.

**Table 2:** Chromatographic condition for analysis of the content of active ingredients of azithromycin.

Column	Mobile phase	Flow rate	Detector	Temp.	Injection vol.	Run time	System suitability parameters considered
RP-C18 column (4.6mmx25cmx5μm)	The mobile phase prepared by filtering and degassing the mixture of acetonitrile, methanol and solution A, in the ratio of 9ml: 3ml: 8ml respectively	1.5ml/min	PDAD; 210nm	50°C	50μl	30 min.	RSD (NMT 2%, Tailing factor(NMT 2.0), and Theoretical plate(NLT 1000)

*Chromatographic systems*. Procedure: An equal volume of about Azithromycin working standard, and the Assay preparation are separately injected into the chromatograph, and the peak area of the responses are measured for all the peaks and compared with USP36/NF31 acceptance limit (% label claim of 90%-110%) Eq (3).


$$\%\text{Content}\;\text{of}\;\text{Azithromycin}=\left(\frac{\text{ru}}{\text{rs}}\right)\times \left(\frac{\text{Cs}}{\text{Cu}}\right)\times \text{P}\times \text{F}\times 100$$ (3)

Where,

Ru = is the peak response of azithromycin Sample solution, Rs = is peak response of the Azithromycin working standard, Cs = is the concentration of USP Azithromycin working standard (mg.ml), Cu = is the concentration of the sample solution (mg/ml), p = is the potency of USP azithromycin working standard,(μg/mg), and F = is conversion factor (0. 001mg/μg)

*Construction of calibration curve*. The concentration of Azithromycin working standard was prepared by dissolving 20mg of working standard into 50 ml volumetric flask. Then, up to the mark, diluent A was added to generate a solution with a known concentration of around 400μg/ml. From this, standard solutions containing about 320, 360, 400, 440, and 480μg/ml were made at a concentration range of 80%-120% as per Food, and Drug Administration office of Regulatory affairs guideline [35]. Then, a volume of around 50 μl was injected into the HPLC. Then, their respective Areas under the Curve (AUC) were recorded. The calibration curve was then determined by plotting the concentration of Azithromycin working standard vs. peak area. The equation for the calibration curve of the Azithromycin working standard was displayed in Eq (4):-

AUC=mX+b (4)

AUC = Area under the curve of the chromatographic peak of Azithromycin, m = Slope of the straight line, b = intercept on the AUC (y axis), and X = concentration of analyte.

#### Dissolution test

*Preparation of diluent*. About 17.5 g of dibasic potassium phosphate is transferred to a 1000ml-volumetric flask and diluted with water to a required volume by adjusting it with a phosphoric acid to a pH of 8.00 ± 0.05. Finally, a mixture of this solution and ACN (80:20) were prepared.

*Preparation of solution A and mobile phase*. Dibasic potassium phosphate about of 4.4g, and 0.5g of sodium1-octanesulfonate were mixed and transferred to a 1000ml of volumetric flask, and diluted with water to a required volume. Then the solution was adjusted with phosphoric acid to a PH of 8.20±0.05. Then, mobile phase was prepared by filtering and degassing the mixture of acetonitrile, methanol, and solution A in the ratio of 9:3:8, respectively.

*Standard solution preparation*. About 50mg of azithromycin working standard was dissolved in 100ml of phosphate buffer to obtain a solution having a known concentration of 0.5mg/ml. To prepare standard solution, first 25ml of standard stock solution was transferred to 50 ml volumetric flask and diluted to a volume with diluent to the required volume to obtain a standard solution having a known concentration about 0.25mg/ml.

*Chromatographic systems*. HPLC is equipped with a 210-nm detector and a 4.6 mm × 15 cm column that contains 5-μm packing L1. Mobile phase was performed by filtering and degassing the mixture of acetonitrile, methanol and solution A, in the ratio of 9: 3: 8 respectively.

*Procedure of dissolution method*. Each of the six vessels of the USP dissolution type 2 apparatus (Paddle method) is filled with 900 mL of phosphate buffer (pH 6.0), and the temperature is maintained at 37 0.5°c and allowed to equilibrate. Once the desired condition was achieved, the tablets of each brand were randomly distributed into each vessel, and then the paddle was rotated at 75rpm. Thirteen milliliters (13 ml) of sample solution were withdrawn using a syringe from the dissolution medium at an appropriate time interval of 5, 10, 20, 30, and 45 minutes and immediately substituted with an equal amount of fresh phosphate buffer to maintain a sink condition. Then, the withdrawal sample was filtered through a 0.45m membrane filter. Then, 11.25ml of the filtrate was transferred to a 25-ml volumetric flask and diluted to the required volume with a diluent (USP 36/NF31) to prepare a sample solution of about 0.25 mg/ml, which is equivalent to the standard solution. Finally, an equal amount of 50μl of sample solution and standard solution were injected into HPLC and the chromatogram output was recorded and calculated by using Eq (5), and compared with the USP acceptance limit.


$$\%\text{Content}\;\text{of}\;\text{drug}\;\text{release}=\left(\frac{\text{Ru}}{\text{Rs}}\right)\times \left(\frac{\text{Cs}}{\text{L}}\right)\times \text{V}\times 100$$ (5)

Where,

Ru = is peak response of azithromycin sample solution (mg/ml), Rs = is peak response of Azithromycin standard solution (mg/ml), Cs = is concentration of standard solution, L = is tablet label claim, V = is the volume of the dissolution medium (900ml) (phosphate buffer).

### Data analysis

The Microsoft Excel-2010, the Statistical Package for Social Sciences version-20 (IBM-SPSS, Chicago, USA), and the KinetDS 3.0 software program were used for statistical and graphical analyses of analytical data obtained from the experimental part of the study. The Microsoft Excel was used for drawing the calibration curve of the working standard’s as well as plotting the graph of the time-dependent dissolution profiles of the drug.

The thickness, diameter, hardness, friability, weight variation, disintegration, assay, and dissolution of different brands were compared by one way ANOVA. Statistical significant differences were considered when P<0.05.

Post-hoc dunnett’s test (p<0.05), model-independent, and model-dependent approaches were used to compare the *in vitro* dissolution profiles of the different brands. The KinetDS 3.0 software program was used to evaluate the release of drug substances from the dosage.

#### Model independent approaches

To compare the dissolution profiles of Azithromycin tablets under study, model-independent methods were considered by applying fit factors (F 1 and F 2), Dissolution efficiency (DE) and Mean dissolution time (MDT).

#### Fit factors

The difference factor (f1) and similarity factor (f2) of all formulations (A1 to A6) were determined to choose the optimum formulation from the tested brands using Eqs (6) and (7). Two dissolution profiles were considered similar and bioequivalent, if f1 is between 0 and 15 and f2 is between 50 and 100 [36].


$$\text{f}1=\{[\overset{n}{\underset{t=1}{\sum }}\left|Rt-Tt\right|]⁄\sum_{t=1}^{n}\left[Rt\right]\}\times 100$$ (6)

$$\text{f}2=50\text{log}\left\{\left[\frac{100}{\sqrt{\left[1+\sum_{t=1}^{n}\left\{Rt-Tt\right\}2/n\right]}}\right]\right\}$$ (7)

Where;

n = is the number of time points, Rt = is the dissolution value of comparator product at time t, Tt = is the dissolution value for the test product.

*Dissolution efficiency*. Dissolution efficiency is the area under the dissolution curve within a time range (t1–t2) [37]. DE was calculated by using the following Eq (8).


$$\text{DE}=\frac{\int_{\text{t}1}^{\text{t}2}\text{y}.\text{dt}}{\text{y}100\left(\text{t}2-\text{t}1\right)\text{X}100}$$ (8)

Where;

y = is the percentage dissolved at time t. The integral of the numerator which is the area under the curve was calculated using the following Eq (9).


$$\text{AUC}=\overset{\text{n}}{\underset{\text{i}=1}{\sum }}\left(\text{t}1-\text{ti}-1\right)\left(\text{yi}-1-\text{yi}\right)/2$$ (9)

Where;

t_i_ = is the i^th^ time point, and y_i_ is the % of dissolved product at time t.

*Mean dissolution time*. Mean dissolution time was also considered in the study to characterize the drug release rate from the dosage form and the retarding efficiency of the polymer [38], and calculated using Eq (10).


$$\text{MDT}=\sum \left[\text{ti}.\Delta \text{Qi}\right]/\text{Q}\infty$$ (10)

Where;

ti, = is an intermediate time of the intervals of sampling time

ΔQi = is the amount of API dissolved in every interval of t”,

Q∞ is the maximum of API dissolved.

#### Model dependent approaches

A Several mathematical models have been proposed to study dissolution profiles in order to decide the kinetics of drug release [39]. The models were evaluated by selecting a certain parameters based on % cumulative drug releases vs. time taken. To understand azithromycin drug release kinetics, various mathematical models dependent approaches were employed (Table 3).

**Table 3:** Mathematical models for comparison of dissolution profiles azithromycin tablets.

Models	Equation
Zero-order kinetic model	Qt = Q0 + K0t
First-order kinetic model	logQt = logQ0 − k1t/2.303
Second-order kinetic model	$\frac{1}{Q}=k.t+\frac{1}{Qo}$
Third-order kinetic model	$\sqrt{Q}=k.t+\surd Qo$
Higuchi kinetic model	Qt = Kh × t½
Weibull kinetic model	*log*[−*ln*(1 − *m*)] = *βlog*(*t* − *Ti*) − *logα*
Hixson-crowell kinetic model	$\sqrt[{3}]{Qo}-\sqrt[{3}]{Qt}=Khc.t$
Korsemeyer-peppas kinetic model	Mt/M∝ = Ktn

Where;

Qt = is the amount of drug released in time t

Qo = is the initial amount of drug

Mt/M∝ = is the amount of drug released at time t/the amount of drug released at infinite time t

m = is accumulated fraction of the drug,

β = is shape parameter,

α = is scale parameter

Ti is location parameter

n is release exponent.

ko, k_1_, kh, khc, and k is release rate constant.

## Results

Six different brands of Azithromycin tablets with a label strength of 500 mg were obtained from a private and hospital pharmacy in Adama and Modjo town, five of which were manufactured in three different countries (India, Turkey, and Greece), and one from a local manufacturer. When the study was conducted, all of the products were within their expiration dates.

### The result of tested Azithromycin tablets

The different brands of Azithromycin tablets under study were evaluated for various physical parameters such as visual inspection, thickness, diameter, hardness, friability, uniformity of the weight, and disintegration time test. From the study, it has been observed that all of the evaluated azithromycin tablets complied with the physical quality requirements.

#### Visual inspection

Six different brands of azithromycin tablets were visually inspected for their consistency in terms of packaging, labeling, and physical characteristics of their tablets. The physical characteristics of the tablets evaluated in the study area revealed that they were free of breakage, cracking, and splitting. The tablets had a uniform shape, color, and markings. Additionally, all brands are free of embedded spots or contamination on their surface. Therefore, none of the tested brands’ packaging, labelling information, and physical attributes showed evidence of being spurious, faked, or fraudulent and agreed with the WHO visual inspection tool (S4 File).

#### Thickness and diameter of test results

The thickness and diameter of all Azithromycin tablet brands tested were determined to be within their allowed limits (±5%) [31] (Table 4). The standard deviation of the thickness and diameter of tested products ranged from 5.3±0.02 to 6.9±0.03mm, and 16.7±0.09mm to 20.2±0.02mm respectively. As a result, all of the tested brands had thickness and diameter values that were deemed to be satisfactory as they were within the acceptable range. However, statistical analysis of the thickness and diameter results from a one-way ANOVA at a 95% confidence interval revealed a significant difference (p<0.05) between the sample mean of thickness, and diameter of all brands.

**Table 4:** Thickness, diameter, and hardness test results.

Code	Thickness (mm) (Mean±SD)		Diameter (mm), (Mean±SD)		Hardness (kpa), (Mean±SD)	
**A1**	6.85±0.03	P = 0.001	18.14±0.02	P = 0.001	17.04±0.2	P = 0.001
**A2**	6.4±0.03		19.12±0.03		17.02±0.3	
**A3**	6.2±0.07		16.7±0.09		17.3±1.8	
**A4**	6.0±0.03		19.2±0.03		20.6±0.8	
**A5**	5.3±0.02		20.2±0.02		27.5±0.6	
**A6**	6.9±0.03		19.7±0.03		15.96±1.1	

#### Hardness test results

The hardness of azithromycin samples were investigated were in the range of 15.96±1.1Kpa to 27.5±0.6Kpa as reported (Table 4). Brand A5 had the highest hardness value (27.5Kpa) while brand A6 had the lowest hardness value (15.9611Kpa). For an acceptable tablet hardness test, a minimum force of approximately 12kpa satisfactory [32]. Hence, all azithromycin brands that were assessed agreed with the hardness test specification. In contrary, the one-way ANOVA (95%) statistical analysis revealed a significant difference (p<0.05) between the sample mean of hardness of all brands in the study.

#### Friability test results

The friability test revealed that all brands of Azithromycin tablets had friability values ranging from 0.004% to 0.04% (Table 5). According to USP40-NF 35, percent friability value of tablets should be less than 1%. Thus, all brands passed the friability specification. Apart from that, there was no evidence of tablet cracking, chipping, or breaking. The one-way ANOVA revealed, there was no a significant difference (p>0.05) between the mean % weight loss of azithromycin brands at the USP specified limit (below 1%).

**Table 5:** Friability, weight variation, and disintegration test results.

Code	% weight loss		Weight in mg (Mean±SD)	Lower bound	Upper bound	P-value	Disintegration(minute) Mean±SD	
**A1**	0.004	P = 0.197	922.95±0.9	921.06	924.8	P = 0.001	4.8±0.3	P = 0.001
**A2**	0.04		889.89±1.8	886.053	893.74		11.8±0.3	
**A3**	0.02		754.79±1.8	751.03	758.54		4±0.5	
**A4**	0.04		925.42±0.001	925.46	925.47		6.8±0.3	
**A5**	0.04		901.3±1.7	888.53	914.003		6.3±0.45	
A6*	0.02		942.5±0.5	941.4	943.61		3.6±0.32	

#### Weight variation test results

According to the specification, the tablet passes the USP uniformity of weight test for drug products with an average strength of >324 mg if no more than two individual weights deviate from the average weight by more than 5% and none by more than 10% (USP36).

According to the study, the weight uniformity of six of the products was in compliance with the Pharmacopoeia specification (Table 5). The one-way statistical analysis result of the variance (ANOVA) at 95% confidence interval (CI) revealed that there was a significant difference (P<0.001) among the sample mean weight of all brands in the study.

#### Disintegration test results

The mean disintegration times of the different brands of Azithromycin tablets were shown (Table 5), all the brands tested complied with the USP40-NF35 specified time for disintegration of tablets which must be within 30 minutes [33]. There is a variation of disintegration time within the sample tablets examined in study.

Brand A6, showed fast disintegration times (3.6±0.32min) while brand A2 (11.8±0.3min) had a long disintegration time. The results of a one-way ANOVA with a 95% confidence interval revealed that there is a significant difference (p<0.05) between the studied azithromycin brands.

#### System suitability of Azithromycin working standard

Six (n = 6) replicate 50μl of working standard solutions were injected into the HPLC to ascertain system suitability parameters. The system suitability was checked by analyzing relative standard deviation, tailing factor (peak symmetry) and theoretical plate.

The system is found suitable (Table 6) in respect of relative standard deviation (%RSD; 0.041), tailing factor (average; 1.104), and theoretical plate (average; 4838) for azithromycin as per USP36 requirements.

**Table 6:** System suitability test results for chromatographic method for Assay test.

Azithromycin Working standard (n = 6)				
Standard injection	Retention time	Peak area	Tailing factor	Theoretical plate
**WS 1**^**st**^ **inj**	6.375	1187320	1.104	4831
**WS 2**^**nd**^ **inj**	6.376	1186679	1.104	4837
**WS 3**^**nd**^ **inj**	6.375	1186792	1.103	4841
**WS 4**^**th**^ **inj**	6.375	1187209	1.105	4850
**WS 5**^**th**^ **inj**	6.375	1187345	1.105	4853
**WS 6**^**th**^ **inj**	6.375	1188043	1.103	4817
**Average value**	6.375	1187231	1.104	4838
**%RSD**	0.006	0.041	0.066	0.273
**Acceptance limit**	NMT 2% RSD	NMT 2%RSD	NMT 2.0	NLT 1000
**Compliance**	Compliant	Compliant	Compliant	Compliant

**Abbreviation: WS**; working standard, **Inj**; injection, **RSD**; Relative standard deviation

The HPLC chromatogram output of the Azithromycin Working Standard for the assay method was nearly the same retention time (S1 Fig).

#### Calibration curve for assay study

The standard of azithromycin was prepared in concentrations of 320, 360, 400, 440, and 480μg/ml. Then, a volume of around 50μl was injected into the HPLC machine, and their respective Areas under the Curve (AUC) were considered. The calibration curves were then generated by plotting Azithromycin working concentrations against peak area. The calibration curve confirmed that, the linear regression equation Y = 3000.2x-10704, where; Y represents the HPLC response (peak area) and X represents the concentration of the working standard. The curve revealed a strong linear relationship (r^2^ = 0.9998) between the concentration of the tested samples and the peak area values (S2 Fig).

#### Identification test result of Azithromycin tablets

The identification test was carried out according to the United States Pharmacopeia, which stated that the retention time (Rt) of the peaks in the chromatogram of all azithromycin samples should be comparable with the respective working standard. According to the study, all of the samples’ retention times were determined to be approximately identical when compared to the standards (Table 7). Hence, the results revealed that all Azithromycin tablets tested had the intended active ingredients.

**Table 7:** Retention time of Azithromycin samples and deviation from the standard.

Code	Average Retention time.(minute) of samples	Average Retention Time of Working Standard	Deviation
**A1**	6.374	6.375	0.001
**A2**	6.376		-0.001
**A3**	6.376		-0.001
**A4**	6.377		-0.002
**A5**	6.375		-
**A6**	6.376		-0.001

#### Assay results of tested azithromycin tablets

An important quality control parameter for optimising drug release is the amount of active ingredient present in a product. All of the samples met the pharmacopoeia limit for Azithromycin tablets, which states that azithromycin must contain 90% to 110% of the labelled amount of the drug (USP36). Furthermore, all products contained Azithromycin within a labelled claim of the specification (Table 8). Sample A4 (Azitro) had the highest percentage content of active ingredient (102.3%), while sample A3 (Azeescot) (98.6%) had the least percentage of drug content compared with other products. The statistical analysis result of the one-way ANOVA revealed that there was a significant difference (P<0.05) in the drug content of the azithromycin brands evaluated in the study.

**Table 8:** Assay test result of Azithromycin tablets.

Sample code	Assay % (mean±SD) (n = 3)	Acceptance limit (USP)	Compliance	P-value
**A1**	101.2±0.12	90%-110%	Compliant	P = 0.001
**A2**	100.7±0.16		Compliant	
**A3**	98.6±0.1		Compliant	
**A4**	102.3±0.3		Compliant	
**A5**	101.8±0.4		Compliant	
A6*	101.23±0.36		Compliant	

#### System suitability result of Azithromycin working standard for dissolution study

The system suitability parameters were gauged in order to verify the system performance of important characteristics under the study, such as the number of theoretical plates, retention time, tailing factor, and relative standard deviation. The method performance parameters were determined by six replicate injections of about 50μl of azithromycin working solution into the HPLC.

Acceptance Criteria: Relative standard deviation for six replicate injections should be NMT 2%, a tailing factor NMT 2.0, and Theoretical plate count NLT 1000. The system is found suitable as per requirements of United States pharmacopeia (Table 9).

**Table 9:** System suitability test results for chromatographic method for dissolution of Azithromycin tablet.

Azithromycin Working standard (n = 6)				
Standard injection	Retention time	Peak area	Tailing factor	Theoretical plate
**WS 1**^**st**^ **inj**	5.541	537517	1.130	5449
**WS 2**^**nd**^ **inj**	5.543	542434	1.127	5451
**WS 3**^**nd**^ **inj**	5.537	535855	1.127	5440
**WS 4**^**th**^ **inj**	5.567	544336	1.103	5371
**WS 5**^**th**^ **inj**	5.537	540179	1.125	5420
**WS 6**^**th**^ **inj**	5.537	543343	1.125	5408
**Average value**	5.543	540611	1.123	5423
**%RSD**	0.21	0.63	0.87	0.56
**Acceptance limit**	NMT 2% RSD	NMT 2%RSD	NMT 2.0	NLT 1000
**Compliance**	Compliant	Compliant	Compliant	Compliant

**Note**: Inj; injection RSD; Relative standard deviation, NMT; Not more than, NLT, Not less than

#### Dissolution test results of Azithromycin tablets

The dissolution test revealed that all six tested brands of Azithromycin tablets passed the single point dissolution test specification as per USP36/NF31 (Table 10). Azithromycin tablets should release the labelled amount by more than 80% at a single time point of 30 minutes. The release rate of the drug at a single time point ranged from 82.67±0.16% to 101.8±0.01% for A2 (zycin) and A3 (Azeescot), respectively. Drug product A3 had the highest percentage of drug release, while brand A2 had the least amount of drug release at a single time point. In this study, the time dependent dissolution profiles of six brands were also evaluated, to provide information regarding bioavailability and batch-to-batch consistency (S3 Fig).

**Table 10:** Dissolution profile result of azithromycin tablet evaluated in the study.

Sampling time (min)	Percent drug released (Mean±RSD)					
	A1	A2	A3	A4	A5	A6
**5**	90.0±0.03	52.2±0.07	68.3±0.05	93.166±0.5	36.3±0.241	73.4±0.166
**10**	93.592±0.13	75.2±0.72	94.18±0.194	97.402±0.3	62.7±0.249	87.8±0.35
**20**	94.436±0.66	79.81±0.2	101.44±0.199	98.55±0.5	90.5±0.394	90.71±0.05
**30**	95.313±0.03	82.67±0.16	101.8±0.01	98.84±0.2	100.7±1.01	97.8±0.475
**45**	96.179±0.04	83.8±0.18	101.82±0.32	100.496±0.1	101.6±0.062	100.01±0.134

#### Dissolution profile comparison

The study has shown that distinct drug release patterns were observed at different time points among the tested azithromycin brands. To confirm whether there is a statistical difference or not between test samples and comparator products in the release profiles of the six different brands, one-way ANOVA (p<0.05) using post-hoc Dunnett’s test was applied. The result showed that there was no significant variation (P>0.05) in their release profiles among the tested samples and comparator product. Therefore, this suggests the presence of azithromycin products that are statistically equivalent with respect to their *in vitro* release profile (Table 11).

**Table 11:** Dunnett multiple comparisons test for dissolution profile of azithromycin tablets tested with comparator.

Tested brands(I)	Comparator (J)	Mean Difference (I-J)	Std. Error	P-value	95% CI	
					Lower bound	Upper bound
**A1**	A6*	3.94440	9.36522	P = 0.122	-21.2	29.2
**A2**	A6*	-15.23600	9.36522		-40.3	10.0063
**A3**	A6*	3.55000	9.36522		-21.6	28.80
**A4**	A6*	7.73400	9.36522		-17.4	32.98
**A5**	A6*	-11.40800	9.36522		-36.7	13.83

In order to ascertain the interchangeability of different brands with the comparator, model independent parameters were applied. Based on the *in vitro* dissolution profile results, brand A2 was not interchangeable with the comparator as the difference factor (f1) was greater than 15% and the similarity factor less than 50% (Table 12). As per requirements, brand A4, and A5 were not equivalent to comparator product as their f2 value is less than 50%. As a result, all the brands, with the exception of brand A1, and A3, cannot be considered as interchangeable with the comparator product (A6) as their f1, and f2 values are far from the accepted limit of the USFDA in comparing the release profiles of a comparator and a test drug [40].

**Table 12:** Comparisons of dissolution profile by model independent parameters.

Sample code	Model independent approach		DE (%)	Difference of DE	MDT
	f1	f2			
A1	4.4	52.5	90.2	-3.1	3.7
A2	16.91	23.42	73.3	13.8	7.53
A3	3.96	54.76	90.7	-3.6	5.6
A4	8.6	38.1	93.8	-6.7	3.73
A5	12.88	29.32	77.7	9.4	1.34
A6*	-	-	87.1	0	7.74

**Note**: A6*;comparator product, DE; dissolution efficiency, MDT; mean dissolution time,

Difference of DE = comparator- tested samples

Furthermore, the dissolution efficiency was also applied to assess the drug release profile and to determine the interchangeability of the different brands. According to the study, except brand A2 (13.4>10), all other generic brands were similar to the comparator product as the dissolution efficiency was less than 10% (Table 12). The study findings meet the dissolution efficiency guideline’s acceptance limit for therapeutic interchangeability (10%) [41]. In a pharmaceutical setting, measuring mean dissolution time is essential for determining the amount of drug ingredient released from the dosage form and the polymer’s retarding performance. Brand A6 had the highest mean dissolution time (7.74), whereas Brand A5 had the lowest mean dissolution time (1.34).

### Dissolution kinetics

All *in-vitro* release test data was fitted to a kinetic equation. The model with the highest Correlation Coefficient (r^2^) value was regarded as the best fit of the release data after fitting mathematical model-dependent approaches to each individual unit of the dissolution data [42]. The drug release data fit well with the Weibull, and Korsemeyer-peppas release models (Table 13).

**Table 13:** Determination of the dissolution release kinetics by model dependent method.

Model dependent parameters	Azithromycin tested brands code						
	Parameters	A1	A2	A3	A4	A5	A6
Zero-order	r^2^	0.8185	0.6710	0.5641	0.7606	0.8389	0.8604
First-order	r^2^	0.8113	0.6309	0.5454	0.7533	0.7729	0.8299
Second-order	r^2^	0.8040	0.5942	0.5279	0.7459	0.6919	0.7976
Third-order	r^2^	0.7967	0.5613	0.5119	0.7386	0.7180	0.7642
Higuchi-model	r^2^	-0.3144	-0.2385	-0.4635	-0.2735	0.8680	-0.8502
Weibull-model	r^2^	0.9817	0.9283	0.8979	0.4980	0.8369	0.5889
Hixson-crowell model	r^2^	0.8137	0.6440	0.5515	0.7557	0.7972	0.8404
Korsemeyer-peppas-model	r^2^	0.9711	0.8897	0.8346	0.9477	0.9695	0.9721

#### Comparison of the price of the tablets, and its quality

According to the study, different brands of Azithromycin tablets were sold at different prices in the pharmaceutical markets of Adama and Modjo town. The comparator product (A6*) of azithromycin appeared to be more expensive than azithromycin brands that were intended to be interchangeable with the comparator brand (36.22$) (Table 14). The brands’ unit/pack prices ranged from 12.22$ to 36.22$. The study found that all six brands of azithromycin tablets tested within official specification. As a result, the variation of price had no effect on the quality of the brands.

**Table 14:** The comparison of the price vs the quality of azithromycin brands.

Code	Brand Name	Country of Origin	Unit/pack	Price/pack (1USD = 52.46ETB)	Percentage	The quality status of the brands in the study
**A1**	Swazi	Indian	10*3	13.34$	10.3%	Passed
**A2**	Zycin	Ethiopia	10*3	12.96$	10.03%	Passed
**A3**	Azeescot	Indian	10*3	15.25$	11.74%	Passed
**A4**	Azithro	Turkey	10*3	34.31$	26.6%	Passed
**A5**	Zithrocin	Indian	10*3	17.16$	13.3%	Passed
A6*	Zithrotel	Greece	10*3	36.22$	28.03%	Passed
**Total USD**	129.24$				100%	

## Discussion

In the study, the physicochemical properties of six different brands of azithromycin tablets collected from the hospitals and privately owned drug retail outlets found in Adama and Modjo towns were evaluated. Individual units were also subjected to model-independent and model dependent approaches to decide the interchangeability and drug release kinetics of the drug substance from the dosage form.

Even if the amount of active ingredient in the drug preparation is correct, this information is not sufficient to assess whether the drug is genuine. Consequently, a thorough examination of the package is essential. The visual examination result revealed that none of the selected brand tablets’ packaging, labelling information, and physical attributes showed evidence of being spurious, falsified, or fraudulent and agreed with the WHO visual inspection tool [30]. In the pharmaceutical setup, visual inspection is a simple and inexpensive technology that is of paramount importance in monitoring the quality of medicines. What’s more, it can provide important guidance to timely recall suspicious batches, revoke marketing authorizations of unreliable suppliers, as well as to protect the public health [43].

The uniformity in thickness and diameter of tablets are necessary for consumer requirements and also for the packaging of tablets. Tablet thickness and Diameter are controlled within ±5% of a standard value [31]. As a result, all of the tested brands had thickness and diameter values that were deemed to be satisfactory as they were within the acceptable range (Table 4). The finding was similar to the report from India [6]. However, statistical analysis of the thickness and diameter results of the one-way ANOVA at a 95% confidence interval revealed a significant difference (p<0.05) detected between the sample mean of all brands. This could be due to granulation properties such as particle size, and particle dispersion, as well as variations in compressive force, die fill, and a lack of strict monitoring of raw material physical qualities and continuous standardisation of upper and lower punch lengths [44, 45]. None conformity to thickness specification can cause packaging problem and consequently increase cost of transportation.

Hardness is an essential criterion in the determination of the ability of the tablets to resist chipping, abrasion or breakage under conditions of storage, transportation and handling before storage. As shown in Table 5, the hardness value of the tested brands ranged from 15.96kpa to 27.5kpa. All tested samples satisfied the minimum requirements of the hardness (12kpa) [32]. However, a statistical study using one way-Anova demonstrates a significant difference in tablet hardness (P<0.05). This may be the type of binders employed, the particle size distribution, moisture content of granules, and compression force used in the formulation potentially affect tablet hardness [45, 46].

The crushing strength of the tablets is not determined solely by their hardness. As a result, the friability test is critical for the tablet to withstand attrition in the package container, which might result in partial powdering, chipping, or fragmentation of the tablets during handling and transportation. According to USP40-NF35 [33] for most tablets weight loss of less than 1.0% is permissible. In the study, the weight loss values for all brands ranged from 0.004% to 0.04%. Similar findings were reported from Indian [6], and Bangladesh [47], all tested brands passed the hardness and friability specification. As a result, it is possible to conclude that the marketed azithromycin brands are mechanically safe and meet the pharmacopeia’s quality control limit. This is the stringent approach that manufacturing companies take to in-process quality control to ensure consistency in the establishment of product quality [48]. There was no significant variation in the mean percentage weight loss of the tablets (p>0.05) according to the statistical analysis of one-way ANOVA.

The weight variation test is a crucial in-process tablet quality evaluation that accurately reflects the equivalent variation in the medication. The tablet passes the test if no more than two individual weights depart from the average weight by more than 5% and none by more than 10%, according to the USP allowable weight deviation limit for tablets with an average weight of 324 mg or more [34]. The findings of the study revealed that six of the Azithromycin tablets gauged have appropriate weight uniformity within the pharmacopoeia standard. Weight variation tests as quality control parameters serve as a pointer to good manufacturing practices maintained by the manufacturers. Inline with this study, similar findings were reported from Bangladesh [47], and Nigeria [49]. Contrary to this study, in India the three tested azithromycin brands far from the weight variation limit [6].

The weight variation data were also statistically compared using a one-way ANOVA with a 95% confidence interval. A significant variation was observed in the mean weight of the brands among the samples (P<0.05).

The cause of the inconsistency between samples could have been due to the formulation conditions, such as mixing, granulation process, including mass production equipment, and the amount of excipients used. Variation in active ingredients can result in toxicity and ineffectiveness, whereas excipients can alter medication release profiles [50]. Furthermore, a change in tablet weight could indicate a change in the API level of the pharmaceutical formulations.

The most essential stage towards a solution for most tablets is disintegration, or the breaking down of the tablet into smaller particles or granules. A tablet that dissolves quickly in saliva is an attractive dosage form and a patient-centered pharmaceutical preparation [51]. The tablets should disintegrate within 30 minutes, according to the USP40 NF35 (2015). All azithromycin tablets complied with USP standards. This result, agreed with a previous study done in Nigeria [52], and Uganda [53], all Azithromycin brands evaluated passed a disintegration time test. Brand A2 had the greatest average disintegration time of 11.8±0.3 minutes among the tablets that met pharmacopeial criteria, while brand A5 had the shortest average disintegration time of 3.6±0.32 minutes. In terms of statistics, a one-way ANOVA with a 95% confidence interval found a significant difference (p<0.05) between the tested brands. These discrepancies in disintegration time may be due to variances in the quality and quantity of tablet excipients (binder, disintegrant, and lubricant) employed, as well as, the compaction pressure used to make the tablets further influences the disintegration of the tablets [54].

Authenticating medications that contain the predicted active ingredients necessitates an identification test on the API in the formulation. Failure to inhibit infection with the incorrect active component results in pathogen proliferation, progression of the underlying diseases, and the emergence of drug resistance [51]. In the study, all the six brands of Azithromycin tablets evaluated passed the USP specification for identification test. All tested samples displayed retention times corresponding with the working standard. As a result, all Azithromycin tablets tested had the active components that were indicated. This is the attributed effect of sound scientific measurement being followed at every stage of the formulation process. However, there is a minimal variance in the retention duration that could be feasible among tested samples to the relevant working standard. This may be due to the interaction of excipients with the column and the fluctuation of the room temperature.

The amount of an active component in a product is a critical quality control for optimizing drug release. Medication must include the active pharmaceutical component at an authoritatively approved amount that provides the desired therapeutic effect in order to be therapeutically effective. Deribe *et al*. (2020) reported that antibiotics with low levels of active pharmaceutical components are used to treat patients, which has significant consequences such as drug resistance, treatment failure, and increasing treatment costs. Incorrect dose adjustment leads to undesirable consequences such as increased morbidity, mortality, treatment costs, and hospital stay days [55]. According to the study, six Azithromycin tablet samples were analyzed; all of the sampled tablets complied with the pharmacopoeia limit for Azithromycin tablets, which states that, azithromycin must have 90% to 110% of the labelled amount of drug (USP36). Comparable results were reported from Nigeria [52]. However, different report from Nigeria (Lagos), and Ghana (Accra), about 71%/25, and 63%/25, failed assay test of an azithromycin tablets and capsules were reported respectively [19]. In the one-way ANOVA, statistical analysis was conducted at 95%CI. There was a significant difference observed in drug content between and within the different brands of azithromycin tablets (P<0.05). This could be attributed to the fact that these samples were from varied manufacturers who may have been performing different manufacturing processes.

Ever since a medicine must dissolve before it can be absorbed, and because the rate at which a drug dissolves from a dosage form generally influences the rate and amount of absorption. As a result the dissolution rate has received a great deal of attention. It’s now thought to be the most sensitive *in vitro* metric that can predict bioavailability. Azithromycin is Biopharmaceutical Classification System (BSC) II drug, low soluble and highly permeable. Therefore, dissolution is the rate-limiting step for the absorption of the drug. So that *in vitro in vivo* correlation could be expected [8]. When given orally, drugs with slow dissolution rates have intermittent and inadequate absorption, resulting in limited bioavailability. Aside from that, when a significant portion of the medicine fails to dissolve, only a tiny amount of API is available for absorption into the systemic circulation, resulting in the failure to produce the targeted therapeutic effect. The unabsorbed portion of the drug, on the other hand, causes the drug’s associated side effects [56].

According to the finding, all of the brands passed a single-point dissolution test and met USP specifications by releasing more than 80% of the product in less than 30 minutes. However, as compared to other brands, brand A2 had the least dissolution profile release. Drugs with a poor dissolution profile may not be available in sufficient quantity or early enough in the plasma to have a therapeutic effect on the target organ or tissue. This is due to the fact that any factor that influences dissolution rate may also influence the rate and extent of drug absorption [57]. Because of the low release profiles(brand A2), local sources may need to improve some practices, and regulatory authorities may need to increase monitoring of the quality of pharmaceutical products manufactured by local industries. Similar results were reported from India [6], and Bangladesh [58]. However, the dissolution result of the finding different from the study done in Belgium [18], and Cameroon [17]. This may be the variation of manufacturing process and the dissolution test parameters.

Valid statistical analysis of the relevant data is clearly a pivotal part of dissolution profile comparisons. The dissolution profiles of different brands were compared by one-way ANOVA (95%CI) using Dunnett’s test. At different time intervals, the post hok dunnett test result revealed no significant difference between the tested samples and the comparator product (p>0.05). The interchangeability of various brands is not guaranteed by statistically equivalent medicinal products. For this fact, model independent approaches should be required to ascertain the exchange of different brands to the comparator. The dissolution profile data shown that all of the tested Azithromycin brands were not considered bioequivalent to the comparator as f1 values were (>15), and f2 values were (<50). All tested samples except A1, and A3 brands, may not be used interchangeably with the comparator product according to specification established by FDA [37]. Similar reports from Philippines [40], and Nigeria [52], all of the tested azithromycin tablets were not interchangeable to the comparator products. This may be related to the values of f1 (difference factor≤15%), and f2 (similarity factor≥50%) which are very sensitive to the number of dissolution time points.

If the difference in dissolution efficiency between the comparator and test products is within acceptable limits, they are considered equivalent (±10). So, it is possible to say that, with the exception of brand A2, all other generic brands were similar with the comparator products as difference of DE is less than 10% [41]. Similar report from Nigeria [49] that evidence as the dissolution efficiency could be a reliable method of predicting the bioequivalence of the generic products.

In the meantime, mean dissolution time was considered to determine the dissolution rate of the drug and the onset of action. To characterize the drug release rate from the dosage form and the polymer’s retarding performance, the mean dissolution time obtained from the accumulative curves of dissolved API as a function of time was used. The result showed that brand A6 had the highest mean dissolution time (7.74) and brand A5 had the lowest mean dissolution time (1.34) compared with the other evaluated brands. As a result, brand A6 may be distinguished by prolonged drug release from the dosage form and a longer onset of action. On the other hand, brand A5 has a higher rate of dissolution, and has a fast onset of action. The higher the MDT value, the better the polymer’s ability to retain medicines and vice versa [38].

Furthermore, the dissolution profiles of evaluated samples were subjected to model-dependent approaches to estimate the kinetics of drug release from the dosage form. The optimal drug release kinetics model was judged to have the highest Coefficient of Correlation (R^2^) [42]. Various kinetic models have been used in various studies to fit the *in vitro* release data and describe the release kinetics. In this study, Weibull, and Korsemeyer peppas kinetic model was determined to be the best fit for the release of drug substances from the dosage forms. Identical report from Bangladesh that evidence as the Korsemeyer-Peppass model was the best model for the release of azithromycin dosage forms [59].

The study sought that different brand of azithromycin tablets marketed at different prices within Adama, and Modjo towns. The price of pharmaceutical products is a critical determinant of access to medicines within affordable prices particularly in countries with a weak public health sector and people paying for drugs out of their own pockets [60]. According to the study, the unit/pack prices of the brands ranged from 12.96$ to 36.22$. The Domestic azithromycin brands(12.96$) were less in price. This demonstrated that the local manufacturer was promoting the affordablility of drug products interms of price. However, from a quality control standpoint, the price differences between the brands had no effect on the quality of products procured from the respective towns.

## Conclusion, and recommendations

The study attempted to assess the physicochemical quality control parameters as well as pharmaceutical equivalence of the Azithromycin 500mg tablets imported and manufactured locally. All the quality control parameters of the different brands were found within the manufacturer methods, United States Pharmacopeia, and the WHO visual inspection tool. The dissolution profiles comparison result of the fit factors indicated that brand A1, and A3 were deemed better brands for interchangeability with the comparator product. With the exception of brand A2 (13.8% >10%), the dissolution efficiency confirms that all are comparable to the comparator product. A model-dependent approach revealed that the drug release data follows the Weibull, and Korsemeyer peppas kinetic models. Also, the study found that price had no effect on the quality of the products.

Overall, all products were found to be as per quality specification with respect to all quality control parameters under study. However, an *in vitro* dissolution study indicated that all evaluated azithromycin products may not be substituted with the comparator product in clinical practice. The study revealed that different brands of azithromycin tablets marketed in Adama, and Modjo town pharmaceutical market have interchangeability issues, which result in a bioequivalence problem. Furthermore, poor quality drugs are a dynamic phenomenon. Therefore, continuous drug quality monitoring is essential for ensuring the safety, efficacy, and quality of pharmaceutical products in the market.

## Supporting information

S1 Fig Chromatogram of Azithromycin working standard. (DOCX)

S2 Fig Calibration curve of Azithromycin working standard. (DOCX)

S3 Fig Time dependent dissolution profiles of Azithromycin tablets (n = 5). (DOCX)

S1 File Mystery shopper training manual. (DOCX)

S2 File Sample collection protocol. (DOCX)

S3 File Visual inspection tool. (DOCX)

S4 File The Packaging and labeling information of the different brands of Azithromycin tablets in the study. (DOCX)
