# Supplementary material for: Quality evaluation of the Azithromycin tablets commonly marketed in Adama, and Modjo towns, Oromia Regional State, Ethiopia
Source: PLoS One. 2023 Mar 2;18(3):e0282156. doi: 10.1371/journal.pone.0282156 (PMC9980786; doi:10.1371/journal.pone.0282156)

**S1Fig:** Chromatogram of Azithromycin Working standard, and Sample


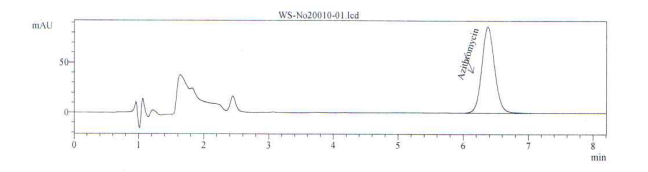


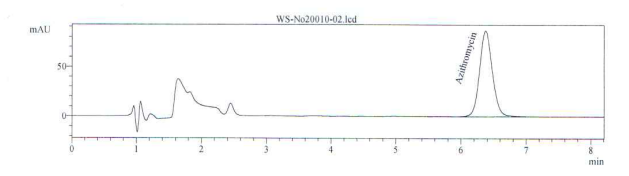

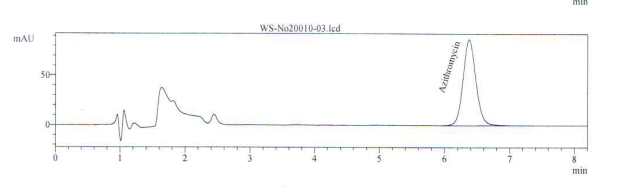


##

## **Peak of Chromatogram of Azithromycin sample**


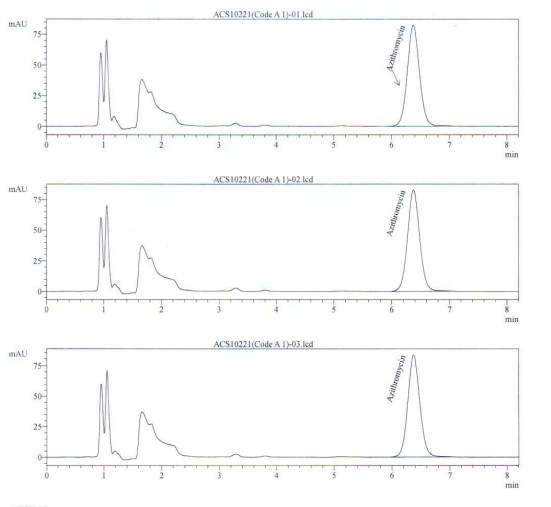

Supplement: S1 Fig — (DOCX) [file pone.0282156.s001.docx]
