# Supplementary figures and images for: Quality evaluation of the Azithromycin tablets commonly marketed in Adama, and Modjo towns, Oromia Regional State, Ethiopia
Source: PLoS One. 2023 Mar 2;18(3):e0282156. doi: 10.1371/journal.pone.0282156 (PMC9980786; doi:10.1371/journal.pone.0282156)

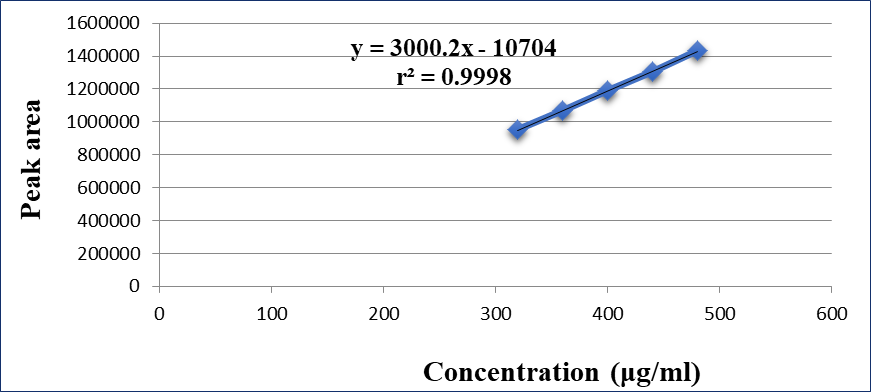


**S2 Fig 1. Calibration curve of Azithromycin working standard**

Supplement: S2 Fig — (DOCX) [file pone.0282156.s002.docx]

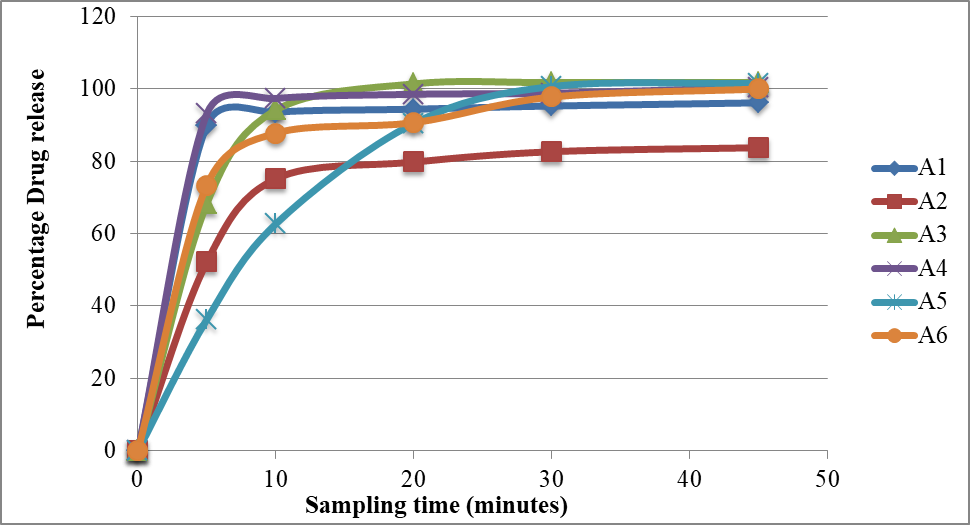


**S3 Fig 3:** Time dependent dissolution profiles of Azithromycin tablets (n=5).

Supplement: S3 Fig — (DOCX) [file pone.0282156.s003.docx]
