# Supplementary material for: Quality evaluation of the Azithromycin tablets commonly marketed in Adama, and Modjo towns, Oromia Regional State, Ethiopia
Source: PLoS One. 2023 Mar 2;18(3):e0282156. doi: 10.1371/journal.pone.0282156 (PMC9980786; doi:10.1371/journal.pone.0282156)
Supplement: S1 File — (DOCX) [file pone.0282156.s004.docx]

## **S1 File. Mystery shopper training manual**

WHO (2015) guidelines on the conduct of the quality of medicines were used to construct mystery shoppers training and selection criteria (manual) on the allocation of medication retail outlets and how to collect available samples for the quality assessment.

**Selection Criteria (Who should sample)**

- The mystery shopper should be able to imitate a "normal shopper" from the community in which the outlet is located, including how to dress and behave appropriately without signs or speech suggesting that they are come from remote area.
- They should be able to use a regular scenario, such as masquerading as a visitor from another part of the country who requires medications for a specific condition, for a specific reason, and for a stereotypical patient.
- Mystery shopper should able to explain the real purpose of the visit to protect him-self/herself in case that his/her identity is revealed, and able to brief the surveyed region after returning from each location to principal investigator, and they should be commitment to complete the assessments and reports on time.

**Why mystery shoppers?**

- Many outlets in countries with weak regulation sell expired, and unregistered medicines, which may outlets staff suspicious, and anxious about the investigation.
- If the seller knows/is concerned that his/her stock contains illegal/poor quality medicines, and that the buyer is potentially linked to the National Medicine regulatory Authority, this may influence which medicine is offered.
- The drug market in many resource-poor nations is highly fragmented, with various marketplaces for people with varied spending power. Even inside a same store, numerous distinct brands of the same medicine will often be available at varying costs, aimed at different market segments. A covert mystery-shopper method may be appropriate in such instances.

**Training for sample collectors**

The mystery shoppers were trained how to sample and approach drug retail outlet staffs when sampling was planned for the study was designed under here.

- They trained was orientated on sampling procedures on how to approach medicine outlets and how to request medicinal products.
- Sample database (containing scanned images or photographs of the dosage form, label, and packaging leaflet), as well as sample recording after leaving the surveyed place they should record details of the purchase;
- Price, name of the provider/outlet, Estimation of temperature at the place should be documented as well as conditions of the purchase, e.g. how many people were in the outlet, how long it took, what was the interaction between the mystery shopper and outlet staff, was it easy to convince the provider to sell medicines, and
- Collected medicines should be properly identified and stored, e.g. in a plastic bag labeled with the name of the outlets.
- The primary investigator for each location will arrange for data collector training to become familiar with the study as well as sample collection guidelines.

**Acknowledgements:**

**Thank you for taking the time to read the training manual that has been provided for you. I hope, you enjoy your time as a mystery shopper with medication retail outlets. If you have any questions about the manual; please don’t hesitate to contact the investigator who assigned you at this task @+251919288056/yetefera19@gmail.com.**
