# Supplementary material for: Quality evaluation of the Azithromycin tablets commonly marketed in Adama, and Modjo towns, Oromia Regional State, Ethiopia
Source: PLoS One. 2023 Mar 2;18(3):e0282156. doi: 10.1371/journal.pone.0282156 (PMC9980786; doi:10.1371/journal.pone.0282156)
Supplement: S2 File — (DOCX) [file pone.0282156.s005.docx]

## **S2 File. Sample Collection Protocol**

Quality Evaluation of Different Brands of Azithromycin Tablets marketed in the selected East Shewa Zone (Adama and Modjo town), Oromia Regional state, Ethiopia.

**Country/Region:_________________________________**

**Place of sample collection: ________________________Sample Code: _________**

**Site of sample collection: ____________________________________**

**Date of Sampling: ______________________________________________________**

**Product name of the sample: ____________________________________________**

Name of active pharmaceutical ingredient(s) (INN): ___________________________

Strength:­­­­­­­­­­­­­­­­­­­­­­___________________________________________________________________

Dosage form: __________________________________________________________

Batch/lot number: _______________________________________________________

Date of manufacture: __________________Expiry date: ________________________

Name and address of the manufacturer: ______________________________________

Country of origin:_______________________________________________________

Registration/license number (if applicable): ___________________________________

Quantity collected (number of tablets, and number of packages):___________________

**Date and Signature of person(s) taking of the samples**

**Name Date Signature**

**1. ______________________________________ _________ ____________**

**2. ______________________________________ _________ ____________**
