# Supplementary material for: Quality evaluation of the Azithromycin tablets commonly marketed in Adama, and Modjo towns, Oromia Regional State, Ethiopia
Source: PLoS One. 2023 Mar 2;18(3):e0282156. doi: 10.1371/journal.pone.0282156 (PMC9980786; doi:10.1371/journal.pone.0282156)

## **S3 File: Visual Inspection Tool**

**EPHARM DQ sample code: _________ Product Name: Azithromycin Tablets.**

A visual inspection checklist for Azithromycin tablets will be set up in order to identify suspect items that will be investigated further. This tool is intended to assist health care professionals in visually inspecting drugs for signs of counterfeiting, such as incorrect packaging, labeling, dosage definition, missing details information regarding dosage, or expiration and manufacturing dates, which should be reported to the appropriate authority. WHO produced the tools that were used for the inspection.

**Packaging:** visual inspection could be an important screening step for product quality control by ensuring the container and closure system, labeling and physical characteristics of the formulation.

| 1. Container and Closure | Yes | No | Other observation |
| --- | --- | --- | --- |
| 1. Do the container and closure protect the drug from the outside environment e.g. properly sealed? 2. Are the container and closure appropriate for the drug insides? 3. Is the container safely sealed? |  |  |  |
| - 1. Labeling |  |  |  |
| 1. If there is a carton protecting the container, does the label on the carton match the label on the container? 2. Is all information on the label legible and indelible? |  |  |  |
| - - 1. The trade name |  |  |  |
| - - - - 1. Is the trade name spelled correctly?         2. Is the drug (trade name) registered in the country by the DRA (drug regulatory authority)?         3. Is the drug legally sold in the country? Does the symbol ® follow the trade name? |  |  |  |
| - - 1. The active ingredient name (scientific name) |  |  |  |
| 1. Is the active ingredient name spelled correctly? 2. Do the trade name and the active ingredient name correspond to the registered drug? |  |  |  |
| - - 1. The manufacturer’s name and logo |  |  |  |
| 1. Are the manufacturer’s name and logo legible and correct? 2. Does the logo or hologram (if applicable) look authentic? 3. Does it change color when viewed from different angles? 4. Is the manufacturer’s full address legible and correct? 5. Has the company or its agent registered the drug in the country? |  |  |  |
| - - 1. The drug strength (mg/unit) |  |  |  |
| 1. Is the strength - the amount of active ingredient per unit - clearly stated on the label? |  |  |  |
| - - 1. The dosage form |  |  |  |
| 1. Is the dosage clearly indicated? 2. Is the indicated drug under this dosage form is registered and authorized for sale in the country? |  |  |  |
| - - 1. The number of units per container |  |  |  |
| 1. Does the number of tablets listed on the label match the number of tablets stated on the container? |  |  |  |
| - - 1. The batch (or lot) number |  |  |  |
| 1. Does the numbering system on the package correspond to that of the producing company? |  |  |  |
| - - 1. The manufacture date and the expiry date |  |  |  |
| 1. Are the manufacture and expiry dates clearly indicated on the label? |  |  |  |
| - - 1. Storage information: |  |  |  |
| 1. Are the storage conditions indicated on the label? 2. Has the drug been properly stored? |  |  |  |
| - - 1. Leaflet or package insert |  |  |  |
| 1. Is the package insert printed on the same colored or same quality paper as the original? 2. Is the ink on the package insert or packaging smudge-proof? |  |  |  |
| 1. Physical characteristics of tablets |  |  |  |
| 1. Are the tablets uniform in shape, size, and texture and marking? 2. Are the tablets free of breaks, cracks, and splits 3. Are there embedded spots/contamination? 4. Does the medicine smell the same as original? |  |  |  |


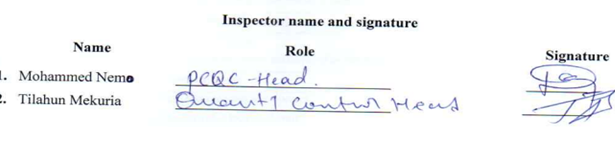

Supplement: S3 File — (DOCX) [file pone.0282156.s006.docx]
