# Supplementary material for: Quality evaluation of the Azithromycin tablets commonly marketed in Adama, and Modjo towns, Oromia Regional State, Ethiopia
Source: PLoS One. 2023 Mar 2;18(3):e0282156. doi: 10.1371/journal.pone.0282156 (PMC9980786; doi:10.1371/journal.pone.0282156)
Supplement: S4 File — (DOCX) [file pone.0282156.s007.docx]

## **S4 File. The Packaging and Labeling Information of the different brands of Azithromycin tablets in the study**

| **Code** | **Packaging** | | | | | | | | | | | |
| --- | --- | --- | --- | --- | --- | --- | --- | --- | --- | --- | --- | --- |
|  | Labeling information | | | | | | | | | | | |
|  | brand name | Active ingredient name | Manufacturer name and logo | Manufacturer full address | Medicine strength (mg/unit) | Dosage form | No.unit/  container | Dosage statement | Batch.No | Mfg. and Exp.date | Storage information | Leaflet/  package insert |
| **A1** |  |  |  |  |  |  |  |  |  |  |  |  |
| **A2** |  |  |  |  |  |  |  |  |  |  |  |  |
| **A3** |  |  |  |  |  |  |  |  |  |  |  |  |
| **A4** |  |  |  |  |  |  |  |  |  |  |  |  |
| **A5** |  |  |  |  |  |  |  |  |  |  |  |  |
| **A6*** |  |  |  |  |  |  |  |  |  |  |  |  |
